# Revision Rates for Osteogenesis Imperfecta Patients Treated with Telescopic Nails. A follow-up Study After a 7-year Experience

**DOI:** 10.25122/jml-2020-0161

**Published:** 2020

**Authors:** Alin Gabriel Sterian, Alexandru Ulici

**Affiliations:** Department of Pediatric Orthopedics, Carol Davila University of Medicine and Pharmacy, Bucharest, Romania

**Keywords:** Osteogenesis imperfecta, Fassier - Duval, revision surgery, telescopic nail

## Abstract

Osteogenesis imperfecta (OI) is a genetically determined systemic pathology that involves a disturbance in the synthesis of type one collagen and is mainly characterized by bone brittleness and other abnormalities. The musculoskeletal system is the most affected by bone fracture after mild mechanical stress occurs. Pathologic bowing appears without trauma, and hyperlaxity is jeopardizing joint stability. Having such an abrupt debut, some patients report in-utero fractures, and treatment is very challenging since surgery has to be postponed until the children grow and become good candidates for intramedullary nailing. In this paper, we discuss the experience of “Grigore Alexandrescu” Hospital for Children in treating these patients and the results we obtained regarding the revision surgeries that we had done. Revision surgery is mandatory for OI patients, and there are multiple factors in deciding to use an implant. We looked back at the data collected for those cases where revision was not related to infection, trauma, or disengagement of the male-female system due to the overlengthening of the implant. The conclusions made are comparable to other centers worldwide with respect to lower limb surgery, and we changed the management protocol according to the problems encountered.

## Introduction

Osteogenesis imperfecta (OI) is a rare inherited pathology that consists of abnormal type one collagen synthesis that affects all structures in the body. The most important and early sign of this pathology is the appearance of fractures after low-energy trauma, progressive bowing of long bones, joint instability, and chronic bone pain. The most used classification is the one created by Sillence that initially had four types [1] and now is expended to more than 15 types [2] thanks to genetic research and new diagnostic criteria. A new nomenclature was published in 2014 in order to simplify the classification [3] and help understand such an intricate pathology. Clinically, all systems in the body are affected, but changes to the musculoskeletal are the most severe; besides, a variable degree of bone brittleness is present. Patients are suffering from severe hyperlaxity, short stature, scoliosis, progressive bowing of the limbs, and chronic bone pain due to continuous microfractures. Other systemic involvement in the case of OI patients like hearing loss, poor teeth health, aortic regurgitation, mitral valve prolapse, tachycardia, malignant hyperthermia, and vascular fragility makes them a real challenge since a multidisciplinary team is necessary. Sometimes, OI can be so severe that lethal forms were described in the literature and are classified as types II, VII, VIII and IX according to the Silence classification, most of the patients dying soon after birth.

The aim of using orthopedic implants is to prevent bone fracture and progressive skeletal deformities. To some degree, bone quality can be improved by the use of bisphosphonates and food supplementation so that chronic bone pain is more tolerable, and the risk of spontaneous fracture becomes lower. Another key element is the prevention of developing bone deformities as they increase the risk of fracture and decreases function and ambulation. Physio and occupational therapy play a vital role in keeping the children active and socially integrated, and a good muscle tone is translated into better bone quality and fewer fractures.

Telescopic implants were first introduced by Bailey and Dubow [4] and later improved by the team at Sheffield Children Hospital [5] in the United Kingdom. Both implants achieved the desired results in preventing severe displacement after fracturing and diaphyseal bowing appeared only after implant failure. The main drawback of both nails was the arthrotomies necessary to insert the T pieces in the epiphysis. The first revolution in telescopic nailing came in 2011 when the Fassier-Duval implant [6] was introduced, and a joint-sparring procedure was used. The main advantage of using telescopic nails consists of the decrease of revision surgery episodes; now, the implant grows at the same pace as the bone and, theoretically, surgery is planned only when the implant becomes too short in relation to the bone. The only indication for revision are infection, implant failure, or migration of the components.

Until the appearance of the telescopic nail systems, the implants used were limited to K-wires, Kuntscher nails, elastic nails, and plates [7, 8]; all of them proved to have the worst prognosis, with high revision rates. The main disadvantage of using non-telescopic implants is that they resolve the problem for a short period until the bone heals. As soon as the bone starts to grow, the implants become shorter, and surgical treatment is necessary to treat specific complications. The most frequent is fracturing just marginal to the end of the implant, followed by bowing of the nonsupport bone segment and implant protrusion through the cortex. The principle of the telescopic nails is very simple as they have two sliding components that are locked only at one end that is passed through the growth cartilage in the epiphysis. As bone grows in length, the two tethered ends of the nail are “pulled”, sliding them apart and elongating the construct. Thus, no segment of the bone remains unprotected, and no residual deformities appear at the ends of the bone. The only problem is encountered when the sliding mechanism stops and the rod becomes too short. On the whole, revision rates are fewer and more spread apart when comparing them to the more classic surgical alternatives, usually appearing for reasons other than a traumatic event.

## Material and Methods

A retrospective review of all the patients diagnosed with OI was carried, and the study group was comprised of 32 patients diagnosed with either type 1 or type 3 OI between 2013 and 2020. All of the patients had surgery at the “Grigore Alexandrescu” Hospital for Children in Bucharest and were operated on by the same team. For all the patients, we used the Fassier-Duval telescopic nail, and the male-female ratio was 2:1. Age ranged from 3 to 15 years, and 17 patients were diagnosed with type 1 and 15 with type 3 OI. The inclusion criteria were: diagnosis of OI, a previous rod placement in either the femur or tibia, currently receiving bisphosphonate treatment and revision performed for reasons other than a traumatic event (displacement or bending of the implant), infection or normal revision because of the implant shortening in relation to the bone.

We used the original surgical technique proposed by PEGA [9] and Fassier-Duval in those cases where percutaneous insertion was possible [10]. For those patients where the deformity was too severe for percutaneous insertion, we used the Soffiled-Millard technique [11] for obtaining correction and stabilizing the fragments until complete bone healing. After surgery, all patients wore a cast for six weeks until physiotherapy was resumed. Regular X-rays were done in the Outpatient Department (OPD) for any signs of secondary displacement and implant failure. The data was collected from the patient’s history folder, the hospital’s radiological database, theatre protocols, and postoperative OPD notes.

Keeping track of the complications encountered, we divided the group into patients with complications at the level of the tibia or femur, male- and female-related complications, and global implant failure.

## Results

From the data analyzed, we observed that 17 (53.12%) out of the 32 patients enrolled required revision surgery for the reasons mentioned above. The tibia was the most susceptible to revision as 11 patients (34%) out of the 32 in total required reintervention. Also, 11 patients (57%) out of 19 that had a tibial surgery required revision. The femur was a lot less prone to complications as only 4 patients (12.5%) required revision from the group of 32 patients and only 4 (14.28%) out of 28 patients with femoral surgery were taken back to theatres for revision. One patient (0.31%) required revision for both the tibia and femur.

Breaking down the complication groups, out of the 11 patients that had revision surgery for the tibia, 10 of them (90 %) had pullout of the distal end of the male component, and only 1 (10 %) had proximal migration of the female component; we defined it as pushout migration. For the femoral revision group, 3 (75%) patients had a proximal displacement of the female component (pushout), and only 1 (25%) patient had a distal pullout of the male component. The patient with combined revision surgery was diagnosed with femoral female pushout and tibial male pullout. The overall distribution of the patients based on the type of OI consisted of 17 (53.2%) patients that were diagnosed with type 1 and 15 patients (46.8%) with type 3. Out of this group, 4 (23.52%) OI type 1 patients and 11 (76.47 %) OI type 3 patients required revision, which showed that OI type 3 patients are more susceptible to revision.

From the data analyzed, we can clearly state that the tibia has a higher rate of revision than the femur without having a correlation between age, sex, open or percutaneous surgery, diameter of the rod in correlation with the size of the bone, and if adjuvant treatments were administered to the patients. The only difference in revision rates was noticed when comparing the type of OI, making type 3 patients more inclined to need revision surgery. Looking back at the interval after which the complication was identified, the proximal displacement of the femoral female component was by far the fastest to be observed overall, the interval ranging between 4 to 6 weeks postoperatively. From the x-rays evaluated, we saw that the femur is the most vulnerable in this regard, although not being the most frequent complication encountered altogether. This comes in opposition to the distal pullout of the male component that is a complication observed later in the postoperative period, roughly 6 months after cast removal, and after complete healing after the osteotomy is achieved. No distal pullout was noticed before complete bone healing. Other revisions were done for combined male and female displacement (1 patient), these being the least frequent.

## Discussion

The advantages of using the Fassier-Duval technique are well known in the literature and there are a lot of centers that are using such implants for patients diagnosed both with OI and other pathologies. The main advantage that separates them from the rest is the insertion technique and the possibility of avoiding arthrotomies, especially in the ankle, where it could prove to be technically difficult with high morbidity rates. Protecting the soft tissue around the joint translates into faster recovery and ambulation of the patients with a better outcome if the range of motion is normal. But as with all implants, they have their limitations, and revision has to be done at some point. Again, literature is abundant in describing the experience of multiple centers and the results they obtained after using the nail. This paper is describing the experience of “Grigore Alexandrescu” Hospital for Children’s and the conclusions that the medical team has drawn after providing healthcare for patients with OI.

Treating these patients requires equal patience both from the patient and the parents; unfortunately, revision is something that all the members involved have to acknowledge and be prepared for as procedures like this are not that rare. Fortunately, there are great options for treating the sequelae of this pathology, and the quality of life has increased dramatically since telescopic implants have developed throughout the years. Before telescopic rods were introduced, revision rates were high since non-telescopic, static implants were overgrown by the bone and early bone deformity, refractures, and metal migration was a common finding. Pediatric orthopedic surgeons must be aware that treating a child with this pathology takes a lot of effort. Understanding how the pathology evolves, depending on the treatment given, is key for a good outcome. The biggest challenge for these patients is the continuous bone growth and the impact it has on the implant used, some of which can easily show their limits, and revision surgery is not something to be done exceptionally. From the data collected, it is clear that tibial revisions were more common than femoral ones for this study group as it is depicted in Table 1, and this is something comparable with other centers that are dealing with this kind of pathology [12, 13]. This idea is very important for future planning, as all the team members must be prepared for treating these patients. Although we revised our technique for rod placement and changed the follow-up protocol, the data still suggests that something else is the cause for these findings, and there is room for improvement regarding both the technique and the implant. In our center, we created two different protocols for follow-up based on the bone that was operated on. From what we learned, the fastest complication to be observed was the proximal displacement of the femoral female component, and we described it as “pushout” since the component is “pushed” proximally by the greater trochanter. It happened immediately before cast removal and before complete healing after the osteotomies, so we increased the number of OPD appointments and performed X-rays every two weeks in the first 2 months, followed up by one X-ray every 30 days for the next 4 months. We noticed that for all of the patients that experienced this complication, migration happened before complete bone healing, and is of great importance to revise the rod as soon as possible since, in this stage, the callus is still abundant, and no remodeling should happen. In case of further delay, the inside of the bone gets obstructed with callus and reinsertion is impossible; the only option feasible is to replace the whole construct with a slimmer nail, thus decreasing stability and rigidity. This can be difficult in bones where multiple osteotomies were done and tissue quality is poor; the risk of fragment displacement and migration is high when trying to change the implant percutaneously through a proximal portal.

**Table 1:** Patients enrolled in the study and the variables collected for the study. All patients had at least one procedure for intramedullary nailing. Revision is checked as “1” for cases where at least one revision was made; “0” means that no revision was done to either the tibia or femur.

Patient criteria	Sex	OI type	Femur	Tibia	Revision femur	Revision tibia
**1**	F	1	1	1	0	1
**2**	F	1	1	0	1	0
**3**	M	1	1	0	0	0
**4**	F	3	1	1	0	1
**5**	M	3	1	1	0	0
**6**	M	1	1	0	0	0
**7**	M	3	1	1	1	1
**8**	M	3	1	1	0	1
**9**	F	3	0	1	0	1
**10**	F	3	1	1	0	1
**11**	F	1	1	1	0	1
**12**	M	1	1	1	0	0
**13**	F	3	1	1	0	1
**14**	F	1	0	1	0	0
**15**	F	1	1	0	1	0
**16**	M	3	1	0	0	0
**17**	F	3	1	1	0	1
**18**	F	1	0	1	0	1
**19**	F	1	1	1	0	0
**20**	M	3	1	1	0	0
**21**	F	1	1	0	0	0
**22**	F	3	1	1	0	0
**23**	F	1	1	0	0	0
**24**	F	3	1	1	0	1
**25**	M	1	1	0	0	0
**26**	F	1	1	1	0	0
**27**	M	1	1	0	0	0
**28**	F	1	0	1	0	0
**29**	M	1	1	0	0	0
**30**	F	3	1	0	0	0
**31**	F	3	1	0	1	0
**32**	F	3	1	0	1	0

F - female; M - male; OI - osteogenesis imperfecta.

For the tibia, from all the revisions done in every bone, we noticed that distal epiphysis pullout is the most common complication of the male component and has the highest incidence for the tibia itself. In comparison with the femur, it appears later, usually after the osteotomies are completely healed and the callus has remodeled. Thus, the follow-up protocol is slightly changed for the femur. In this situation, we see the patients in the OPD every month for an x-ray checkup and every 2 months after cast removal in the first 6 months after the operation. The difference for this complication is that once the nail is displaced, there is no option for reinsertion, like there is for the female component, so the implant has to be changed completely. In our center, this is done when deformity appears distal to the end of the nail, and the risk of fracturing is high, or the nail protrudes through the cortical wall. If bone density is good and the patient does not skip the bisphosphonate treatment, surgery could be delayed and planned accordantly.

Other complications, like combined male and female displacement, are very rare and require whole implant replacement as soon as possible due to the high risk of deformity and fracturing. There is no acceptable waiting interval since the whole nail is compromised, and both proximal and distal ends of the bone are at risk of fracturing.

## Conclusions

Surgery for OI patients is challenging, and the Fassier-Duval telescopic nail system, although it gave new perspectives and became the most used implant in the world for this pathology, has its limitations and drawbacks. Comparing it to other implants, the failure/revision rate is lower, and the implant is more gentle with the soft tissue in regard to the technique. But there are complications that do arise after using this technique, and there is room for improvement of future telescopic systems. Focusing on the component locking to avoid pullout or pushout of the threads in the epiphysis could be the next step in telescopic nail implants as today’s variants are technically difficult and have questionable results. Overall, it is much cheaper and less stressful to use such an implant rather than going for non-telescopic implants that have high revision rates that puts a lot of stress on the patients and their families, but the physicians involved must know that there is no “revision proof” implant on the market and they have to plan ahead. Surgery for revising such implants can be challenging if not done at the right time, and the follow-up protocol must be tailored for each patient individually, so the transition from one implant to another is as easily tolerable as possible.

## Conflict of Interest

The authors declare that there is no conflict of interest.
